# Draft genome sequence of *Dermacoccus nishinomiyaensis* Y5

**DOI:** 10.17912/micropub.biology.002155

**Published:** 2026-08-12

**Authors:** Jewelia T Keller, Shen Jean Lim, Ojas Natarajan, Nicholas Cropper, Larry J Dishaw, Mya Breitbart

**Affiliations:** 1 University of South Florida, Tampa, FL, US; 2 College of Marine Science, University of South Florida, St. Petersburg, FL, US; 3 College of Arts, Science and Education, Florida International University, Miami, FL, US; 4 Department of Pediatrics, Morsani College of Medicine, University of South Florida, Tampa, FL, US; 5 Department of Integrative Biology, University of South Florida, St. Petersburg, FL, US

## Abstract

We report the draft genome sequence of *Dermacoccus nishinomiyaensis* Y5 isolated from a glycerol stock prepared from a fungal culture from the gill of the lucinid bivalve, *Stewartia floridana* . This bacterial strain is gram-positive, coccus-shaped, and citrate-positive. Its draft genome of 3.3 Mb was assembled with 100% completeness, comprising 11 contigs and 2,937 protein-coding genes. *Dermacoccus nishinomiyaensis* Y5 shared 98.9% average nucleotide identity (ANI) with its closest genome relative, *D. nishinomiyaensis* CTOTU46710 assembled from an urban metagenome. Further investigation is needed to identify the source and pathogenicity of *D. nishinomiyaensis* Y5.

**Figure 1. Average Nucleotide Identity (ANI) comparisons across Dermacoccus nishinomiyaensis genomes:** Average Nucleotide Identity (ANI) comparisons between the genome of *Dermacoccus nishinomiyaensis* Y5 isolated in this study (annotated with black square) and all other representative genomes at the time of analysis assigned to *Dermacoccus nishinomiyaensis* . RefSeq accession numbers are listed in the x-axis and y-axis.

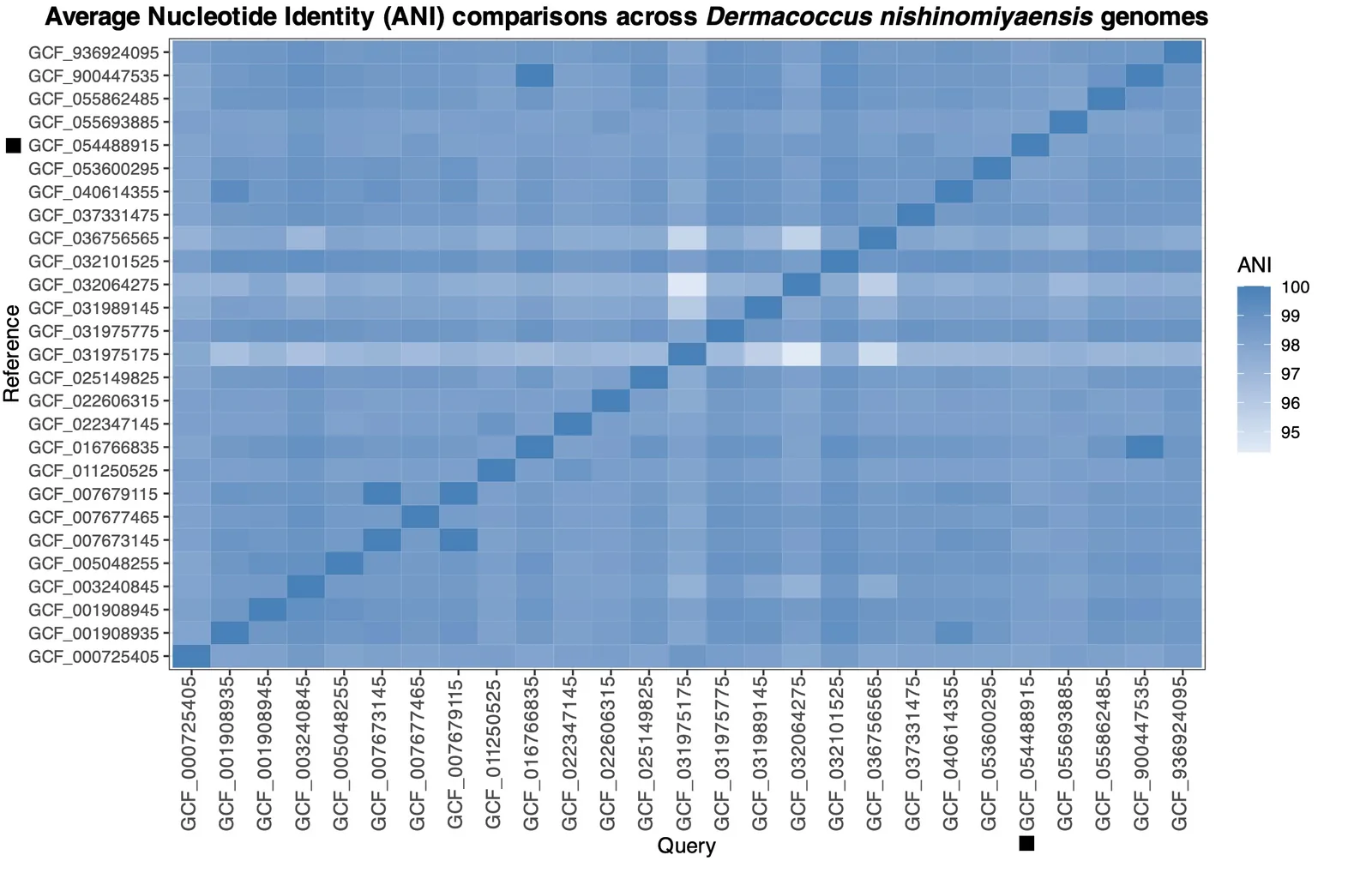



## Description

*Dermacoccus nishinomiyaensis* is a member of the Dermococcaceae family and commonly resides in the skin microbiome, functioning as a skin commensal (Williams and MacLea, 2019). Current literature classifies *D. nishinomiyaensis* as a coccus-shaped, non-spore forming, aerobic, chemo-organotrophic bacterium (Stackebrandt et al., 1995). *Dermacoccus nishinomiyaensis* was isolated serendipitously from a glycerol stock originally prepared from a fungal culture isolated from the gill of the lucinid bivalve, *Stewartia floridana* . The live lucinid (specimen ID: SS1) was collected from the Terra Ceia Aquatic Preserve (GPS coordinates: 27.587404, -82.623248) on September 7, 2023 (Lim et al., 2025).

Dissection and culturing methods were as described in Lim et al., (2025). Briefly, one gill was dissected from the live lucinid specimen, rinsed, homogenized, and diluted 10X (v/v) in 0.2 μm-filtered 35 g/L Instant Ocean® solution (https://www.instantocean.com). Sterile glass plating beads were used to spread 200 μL of the homogenate on BD Difco™ Marine Agar 2216, which was then incubated in the dark at room temperature (~22°C). Fungal growth was observed on the plate after two days and re-inoculated in BD Difco™ Marine Broth 2216 under the same conditions. Upon visible growth, 500 μL of the fungal culture was added to 500 μL of 50% glycerol and stored at -80ºC. The glycerol stock was streaked on BD Difco™ Marine Agar 2216. Instead of fungal growth, yellow colonies indicative of bacterial growth were observed.

One isolated yellow colony (Y5) was randomly chosen for subculturing and maintenance on BD Difco™ Marine Agar 2216. For biochemical characterization, the subculture was inoculated in various media at 37ºC. Under the microscope, this bacterial strain was observed to be gram-positive and coccus-shaped. Based on the phenol red broth test, *D. nishinomiyaensis* Y5 was unable to ferment glucose, lactose or sucrose. The citrate utilization test showed that the strain was citrate-positive. The Sulfide-Indole-Motility (SIM) test indicated the bacterial strain to be non-motile and unable to produce sulfur and indole. Gelatin hydrolysis, urease and phenylalanine deaminase tests produced negative results.

For genome sequencing, three representative yellow colonies maintained on BD Difco™ Marine Agar 2216 were separately grown in BD Difco™ Marine Broth 2216. As described in Lim et al. (2025), DNA was extracted from each culture upon visible growth by centrifuging each culture at 1,700 RCF for 5 minutes and collecting the pellet. DNA extraction was performed using Qiagen's AllPrep DNA/RNA Mini kit, followed by DNA purification using ZR's Genomic DNA Clean & Concentrator-10 kit. The concentration and quality of each purified DNA sample was assessed using the Qubit DNA HS assay, the Nanodrop ND-1000 spectrophotometer, and electrophoresis on a 1% (w/v) agarose gel. The unsheared DNA sample with the highest concentration and quality was sent to Eurofins Genomics for library preparation using Oxford Nanopore Technologies' (ONT) Rapid Barcoding Kit 96 V14. The library was sequenced on a GridION R10.4.1 flow cell and ONT's Dorado v0.6.0 was used for base-calling with the dna_r10.4.1_e8.2_400bps_hac@v4.2.0 model. A total of 66,806 reads and 108,590,115 bases were sequenced with a N50 value of 2,813 bp.

Sequence data was analyzed on the KBase server (Arkin et al., 2018) using default software parameters. Quality filtering by Filtlong v0.2.1 (https://github.com/rrwick/Filtlong) retained 35,588 reads, which was assembled using Flye v2.9.4 (Kolmogorov et al., 2019). The quality of the genome assembly was assessed using Quality Assessment Tool for Genome Assemblies (QUAST) v4.4 (Gurevich et al., 2013). The assembly, totaling 3,301,037 bp, comprised 11 contigs, with 68.78% G+C content and a N50 value of 2,066,152 bp. CheckM v1.0.18 (Parks et al., 2015a) predicted the assembly to be 100% complete with 0% contamination. Using the Genome Taxonomy Database (GTDB) release R226 (Parks et al., 2022b) as reference, GTDB-Tk v2.4.0 (Chaumeil et al., 2022) classified the genome as *Dermacoccus nishinomiyaensis* . The genome shared 98.9% average nucleotide identity (ANI) with the genome of *D. nishinomiyaensis* CTOTU46710 assembled from an urban metagenome (Magnúsdóttir et al., 2023), as calculated by fastANI v1.33 (Jain et al., 2018) (Figure 1). The genome was annotated using the NCBI Prokaryotic Genome Annotation Pipeline v6.10 (Li et al., 2021), which identified 2,937 protein-coding genes and three copies of the 16S rRNA gene. Sequence data, including raw reads (Sequence Read Archive accession: SRX31722345 ) and the genome assembly (RefSeq accession: GCF_054488915) is deposited at NCBI under the BioProject accession number PRJNA1399496 .

To confirm whether the gills of lucinid bivalves contain *D. nishinomiyaensis* Y5, primers 79F (5'-AGCTTGCTGGTGTGGATTAG-3') and 169R (5'-GGAGATCGGTCGTATCCAGTAT-3') targeting the 16S rRNA gene of *D. nishinomiyaensis* Y5 were designed using the Integrated DNA Technologies (IDT) PrimerQuest™Tool (https://www.idtdna.com/pages/tools/primerquest). PCR was performed on the DNA sample extracted from the other gill of the same *S. floridana* specimen using Qiagen's AllPrep DNA/RNA Mini kit, as well as 13 other DNA samples extracted from clam gills dissected from other *S. floridana* specimens listed in Lim et al., (2025) under the following thermocycling conditions: 95ºC for 10 minutes, followed by 34 cycles 95ºC for 30 minutes, 52.5ºC for 30 minutes, 72ºC for 1 minute, and an extension period of 10 minutes at 72ºC. No amplification was observed in all DNA samples tested.

*Dermacoccus nishinomiyaensis* is a Gram-positive, non-spore forming bacterium commonly found as a commensal resident in the human skin microbiome (Stackebrandt et al., 1995). Outside of the skin microbiome, *D. nishinomiyaensis* has been isolated from human-built environments, such as wood ash (Williams and MacLea, 2019) and indoor track facilities (Klein et al., 2017). In the natural environment, *D. nishinomiyaensis* has been isolated from the phycosphere of red algae collected from an intertidal zone in Korea (GCF_055693885; 98.2% ANI with *D. nishinomiyaensis* Y5), the Ulu Slim Hot Spring in Malaysia (GCF_000725405; 98% ANI with *D. nishinomiyaensis* Y5), and the Styx glacier in Antarctica (GCF_ 037331475; 98.7% ANI with *D. nishinomiyaensis* Y5) (Kim et al., 2025). Although the negative PCR results suggest that *D. nishinomiyaensis* Y5 could be a transient contaminant during cultivation and glycerol stock preparation, the gill origin of this bacterium could not be excluded without further testing. Species within the *Dermacoccus* genus do not typically exhibit pathogenic properties; however, it has been documented that *D. nishinomiyaensis* may cause catheter-related infections (Joron et al., 2019). Further investigation is necessary to determine the ecological origin of *D. nishinomiyaensis* Y5 and whether it represents a transient contaminant or a rare marine bacterium.
